# Socio-demographic and clinical predictors of outcome to long-term treatment with lithium in bipolar disorders: a systematic review of the contemporary literature and recommendations from the ISBD/IGSLI Task Force on treatment with lithium

**DOI:** 10.1186/s40345-020-00203-3

**Published:** 2020-12-16

**Authors:** Diane Grillault Laroche, Bruno Etain, Emanuel Severus, Jan Scott, Frank Bellivier, On behalf of the ISBD-IGSLI Joint Task Force on Lithium Treatment

**Affiliations:** 1 INSERM U1144 – Optimisation Thérapeutique en Neuropsychopharmacologie, Université de Paris Descartes, Paris, France; 2 AP-HP, DMU Neurosciences, GH Saint-Louis – Lariboisière – F. Widal, Hôpital Fernand Widal, Département de Psychiatrie et de Médecine Addictologique, Paris, France; 3 Faculté de Médecine, Université de Paris, Paris, France; 4 Centre for Affective Disorders, Institute of Psychiatry, Psychology and Neurosciences, London, UK; 5 Department of Psychiatry and Psychotherapy, University Hospital Carl Gustav Carus, TU Dresden, Dresden, Germany; 6 Academic Psychiatry, Institute of Neuroscience, Newcastle University, Newcastle, UK

**Keywords:** Bipolar disorder, Lithium, Response, Prediction, Mania, Depression, Treatment, Psychiatry, Clinical markers, Predictors

## Abstract

**Objective:**

To identify possible socio-demographic and clinical factors associated with Good Outcome (GO) as compared with Poor Outcome (PO) in adult patients diagnosed with Bipolar Disorder (BD) who received long-term treatment with lithium.

**Methods:**

A comprehensive search of major electronic databases was performed to identify relevant studies that included adults patients (18 years or older) with a diagnosis of BD and reported sociodemographic and/or clinical variables associated with treatment response and/or with illness outcome during long-term treatment to lithium (> = 6 months). The quality of the studies was scored using the Quality Assessment Tool for Observational Cohort and Cross-Sectional Studies from the National Institute of Health.

**Results:**

Following review, 34 publications (from 31 independent datasets) were eligible for inclusion in this review. Most of them (n = 25) used a retrospective design. Only 11 studies were graded as good or borderline good quality. Forty-three potential predictors of outcome to lithium were identified. Four factors were associated with PO to lithium: alcohol use disorder; personality disorders; higher lifetime number of hospital admissions and rapid cycling pattern. Two factors were associated with GO in patients treated with lithium: good social support and episodic evolution of BD. However, when the synthesis of findings was limited to the highest (good or borderline good) quality studies (11 studies), only higher lifetime number of hospitalization admissions remained associated with PO to lithium and no associations remained for GO to lithium.

**Conclusion:**

Despite decades of research on lithium and its clinical use, besides lifetime number of hospital admissions, no factor being consistently associated with GO or PO to lithium was identified. Hence, there remains a substantial gap in our understanding of predictors of outcome of lithium treatment indicating there is a need of high quality research on large representative samples.

## Background

Bipolar disorder (BD) is a highly prevalent psychiatric disorder. With the combination of an early age of onset (peak 15–25 years) and a recurrent course, it is one of the most burdensome disorders worldwide, being ranked 4th in terms of DALYs (disability-adjusted life-year) for all medical conditions in individuals aged less than 25 years, and 6th in working age adults (Gore et al. 2011; Collins et al. 2011). BD is a leading cause of increased premature all-cause mortality including not only suicide, but also medical conditions and, compared with the general population, the estimated decrease in life expectancy is about 10 years (Kessing et al. 2015).

Mood stabilizers are the mainstay of prophylaxis for BD and lithium (Li) is the most frequently recommended first-line treatment in Clinical Practice Guidelines (CPG) (e.g. the National Institute for Health and Clinical Excellence (NICE) 2020 (https://www.nice.org.uk/guidance/cg185); the British Association for Psychopharmacology (BAP) (Goodwin et al. 2016); the Canadian Network for Mood and Anxiety Treatments (CANMAT) (Yatham et al. 2018), Royal Australian and New Zealand College of Psychiatrists (Malhi et al. 2018)). Furthermore, a network meta-analysis comparing the efficacy and tolerability of several classes of mood stabilizers concluded that a particular advantage of Li is that it prevents mood episode relapses and recurrences of all polarities (Miura et al. 2014). Despite evidence of efficacy and support for the use of Li in CPG recommendations, prescribing of Li has plateaued (Kessing 2019) and in many countries patients’ and clinicians’ preference for Li is lower than expected (Tondo et al. 2019). Indeed, antidepressants, despite not recommended as monotherapy or first line use, remain a widely used therapy (Kessing et al. 2016) and other agents such as atypical antipsychotics have been aggressively marketed as alternatives. Another reason for this apparent ambivalence is that a substantial proportion of individuals (probably between 30 and 60%) do not achieve a good outcome with Li prophylaxis in day-to-day clinical settings (Scott et al. 2018). Furthermore, predictors of poor outcome are poorly understood or there is limited evidence supporting their validity. As such, patients are often faced with a ‘trial and error’ approach that requires their commitment to take Li for about several months in order to determine the magnitude of their individual response. Unsurprisingly, this scenario, plus the narrow therapeutic window (requiring plasma monitoring), is associated with high attrition rates and suboptimal exposure to Li treatment, e.g. due to fluctuating adherence, in individuals with BD.

To avoid lengthy and ineffective treatment trials in individuals with a low likelihood of benefit from Li, a number of studies aimed to identify socio-demographic and clinical predictors of good prophylactic response to lithium. Some reviews and meta-analyses have been undertaken to try to summarize findings from a broad range of individual studies (Severus et al. 2014; Kessing et al. 2018). However, both approaches have encountered problems. For example, reviews published in the last 15 years have rarely followed current guidelines on systematic reviews and/or the reported findings show significant inconsistencies across the reviews (Tighe et al. 2011; Rybakowski 2014; Kleindienst et al. 2005; Montlahuc et al. 2019). Likewise, the use of meta-analytic approaches that try to quantify the relative importance of potential predictors of good or poor response to Li are undermined by the fact that many of the original studies are of low quality and so there are significant differences in the estimated importance (and valency of effect) of many factors. For instance, a recent meta-analysis by Hui and colleagues (Hui et al. 2019) identified six predictors of good response (Manic Depressive Interval (MDI) sequence, absence of rapid cycling, absence of psychotic symptoms, family history of BD, shorter duration of illness prior to the start of lithium, and later age of onset). However, findings of large and significant associations between these identified factors and good response (e.g. MDI sequence had an odds ratio of about 4.3) were undermined by the fact that < 10% of the 71 publications included in the systematic review and meta-analysis were rated as good quality. For example, in the case of MDI sequence–one of the core features of the classical manic-depressive clinical profile associated with lithium response by Mogens Schou and others in 1970’s (Schou 1982)–five of the six studies included in the pooled analysis were rated as poor quality which highlights the need to be very cautious in interpreting the relevance of this potential predictor. Indeed, several guidelines suggest that it is inappropriate to undertake meta-analyses under these circumstances or that the use of this approach requires justification (Thompson et Pocock 1991; NIH Quality Assurance Guidelines 2019). On the other hand, in case of MDI sequence, the sixth study in the afore mentioned meta-analysis (Hui et al. 2019), which was considered of fair quality (Maj et al. 1989), also showed that MDI sequence, compared to DMI sequence, was associated with good outcome to lithium treatment.

From a methodological point of view, to be able to truly identify clinical or socio-demographic predictors of outcome to prophylactic treatment with optimal lithium levels a 3-arm randomized placebo- and “active comparator”- controlled study design would be needed—to differentiate between predictors of “treatment versus no treatment” and “treatment with lithium versus treatment with another approved drug”. However we are not aware that any such study has been conducted. Therefore, for this review, we decided that it is more prudent to use the wording “predictors of outcome to long-term treatment with lithium” instead of “predictors of response to long-term treatment with lithium” when it comes to interpret the results of the vast majority of the studies conducted so far.

Given the above, the International Society of BD (ISBD) Task Force on the treatment with Lithium concluded that a gap in the knowledge-base about Li response still remained. Further, it was agreed that this gap might best be addressed by a qualitative review (i.e. design and quality of available studies) that adhered to established guidelines on systematic reviews and that employed a protocol that had been subjected to peer-review (via a recognized international body) and was published (to allow external scrutiny). It was agreed that the review would focus on contemporary publications and that eligibility criteria for inclusion would stipulate the need for any study to provide clear documentation of the procedure for diagnosing BD and for defining Li response, etc. The key aims of the review were:

- To identify socio-demographic and clinical factors that are associated with good outcome as compared with poor outcome to Li prophylaxis in adults with BD;
- to determine if the list of putative predictors remained robust when considering number of studies identifying each specific factor, study quality (good versus fair/poor) and design (prospective versus retrospective).

Due to the known weaknesses in the literature, quantitative analyses were disregarded pre-hoc, but instead a range of qualitative techniques were utilized to assess the above concepts.

## Methods

### Protocol

**Fig. 1:**
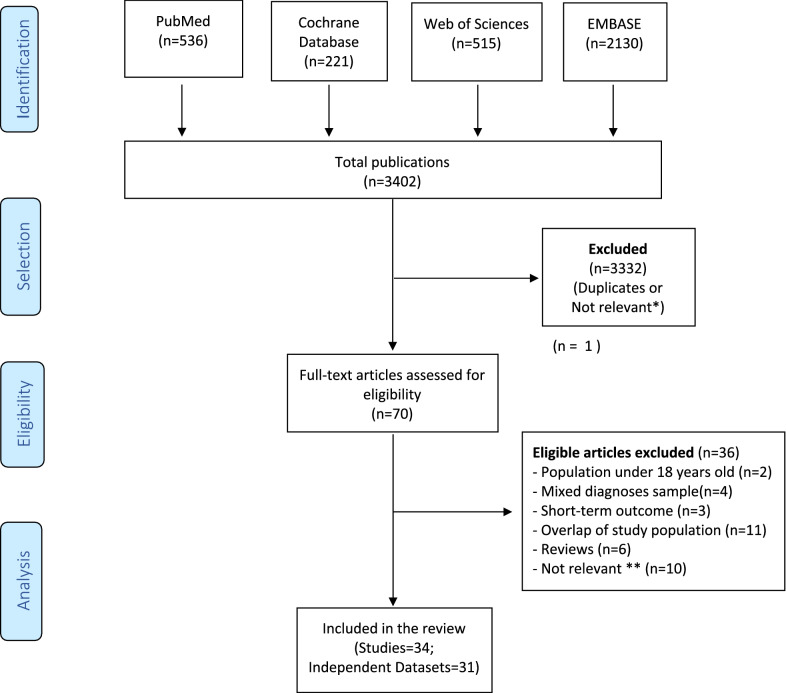
Flow-chart of articles included in the systematic qualitative analysis. (*) not relevant: keywords met selection criteria, but publication did not represent a study of outcome with Lithium. (**) not relevant: studies failed to meet inclusion criteria regarding predictors or met ≥ 1 exclusion criterion e.g. comparison of outcome to Li versus other mood stabilizers

The protocol for the systematic review was lodged with the international prospective register of systematic reviews in July 2019 (PROSPERO: CRD42019141329) and we adhered to the Preferred Reporting Items for Systematic Reviews and Meta‐Analyses (PRISMA) and Meta‐Analysis Of Observational Studies in Epidemiology (MOOSE) guidelines (Liberati et al. 2009; Moher et al. 2015). A PRISMA flowchart is provided in Fig. 1 and a PRISMA checklist is provided in the Additional file 1: (Appendix 1).

### Search strategy

A systematic strategy was employed to search for studies relevant to the review. We employed search terms referring to socio-demographic and clinical variables that may predict response to or can be associated with outcome during long-term exposure to Li in adults with a presentation that met diagnostic criteria for BD. For example, terms such as lithium OR prophylactic lithium AND bipolar dis* AND predict* OR respons* OR remission OR Retrospective Assessment of Response to Lithium Scale OR Alda scale OR treatment outcome OR non-response OR relapse OR remission OR recurrence were cross-referenced with terms identifying observational, retrospective, prospective, cohort, case control, epidemiological and/or database studies, and randomized or non-randomized trials. Searches were repeated using the terms affective psychosis or manic depression related terms (mani*, manic depress*, hypomani*) instead of bipolar dis* and using terms that ensured we focused on adult-pattern disorders (i.e. excluding paediatric/juvenile BD). Also, we incorporated selected search terms used in previous systematic reviews of lithium response and prediction of outcomes for individuals (e.g. Li blood level, etc.).

Electronic databases (MedLINE, PubMed, Cochrane Library, Web of Sciences, EMBASE, and Google Scholar) were searched for the last 30 years (from July 1st 1989 until July 31st, 2019). This time frame was chosen to ensure we focused on more recent research and studies that primarily employed diagnostic criteria for BD that show greater consistency across current classification systems (DSM-IIIR onwards and/or equivalent iterations of ICD). Alerts were set up until December 2019 for the electronic databases so that researchers were notified of new publications up to and including the submission date for this review (e.g. manuscripts listed as Epub ahead of print). A handsearch of primary and secondary publication reference lists was conducted to identify further studies.

### Selection Criteria

Publications were eligible for screening if they were written in English and reported data from peer-reviewed studies in samples that comprised of adults (minimum age 18 years) who met internationally recognized diagnostic criteria for BD (DSM-IIIR and later, ICD9 and later, RDC) and that reported sociodemographic and/or clinical variables associated with treatment response and/or with illness outcome during long-term exposure to Li. Lithium could be used alone, in combination with other psychotropics and/or co-prescribed with another mood stabilizer.

### Eligibility

We selected only those articles reporting sociodemographic and/or clinical factors associated with good outcome to Li treatment compared with poor outcome to Li treatment in adults with a presentation of BD that met internationally recognized diagnostic criteria according to the Diagnostic and Statistical Manual (DSM IIIR or later), the International Statistical Classification of Diseases and Related Health Problems (ICD-9 or later; World Health Organization, 1978; ibid 1992), or the Research Diagnostic Criteria (RDC) (Spitzer et al. 1978). To be included in the systematic review, publications had to fulfill the following eligibility criteria:

### Inclusion criteria

(i) the article clearly indicated that Li was prescribed as a long-term, continuation, maintenance or prophylactic treatment (if the publication did not use any of these descriptors, it could be included if the reported mean duration of Li treatment was at least 6 months),
(ii) ‘outcome’ was defined as any outcome in terms of mood episodes or illness course or activity, but did not include outcomes that were related only to the anti-suicidal properties of lithium,
(iii) raw data about potential socio-demographic or clinical predictors of Li response (measured as a categorical or continuous variable) or of outcomes associated with exposure to Li (e.g. social functioning) were reported,
(iv) findings were reported separately regarding good outcome or poor outcome to Li (where Li was prescribed as a single or combined treatment),
(v) studies where a subsample of the study population met the eligibility criteria could be included in the review provided relevant data were available for that subsample or could be obtained from the authors.

### Exclusion criteria

(i) comparative studies that only reported predictors of outcome to Li versus predictors of outcome to other mood stabilizers (unless the publication separately reported factors specifically associated with good versus poor outcome to Li),
(ii) studies of samples that were wholly or partly comprised of children or adolescents and/or of mixed diagnoses such as BD with unipolar disorder and/or schizo-affective disorders (unless the analyses were stratified according to age groups or to diagnosis, and findings reported separately),
(iii) studies that only reported outcomes for Li treatment in BD samples with an acute manic or depressive episode,
(iv) studies that reported only on putative biomarkers of good or poor outcome to lithium treatment (such as genetic or brain imaging research) without data on socio-demographic or clinical variables associated with good or poor outcome to lithium,
(v) studies where the sample comprised only of a ‘special population’ or subgroup (e.g. seasonal affective or post-partum disorders, or BD in individuals with learning difficulties or with a particular medical disorder such as thyroid disease),
(vi) review articles or non-data based publications.

A priori, we agreed upon a strategy for determining study eligibility when several publications arose from the same database or when publications included overlapping samples. The consensus was that later publications were eligible for inclusion if they included novel findings e.g. a later follow-up had revealed additional possible predictors of outcome to Li treatment (in addition to the initial report). If data were reported separately for repeated follow-ups of the same sample, then we would prioritize findings from the most recent publication with the longest follow-up. If the publications reported overlapping samples, then we would prioritize findings from the largest sample size.

Data publications that included the key search terms in the title, abstract, or index term fields were screened, and full text articles obtained as appropriate. Duplicate publications were removed and uncertainties regarding eligibility of individual articles were reconciled by consensus (by DGL, BE, and checked independently by JS).

### Data extraction

Data were extracted from studies meeting selection criteria and key information was entered into an Excel file proforma (by DGL and BE; then checked independently by FB). This recorded key study details e.g. authors, study location, design (cross-sectional; cohort; retrospective; prospective), and year of publication and noted whether the publication was included in a series of publications by the same research group of investigators and/or whether the study arose from a project being undertaken by a larger network or consortium, etc. Also, the proforma collated specific information related to the review topic, namely sampling frame (sample size, diagnostic assessment procedure); method for assessing Li response (e.g. clinical judgement, self- or observer-rated scale, change in social functioning or episode frequency etc.); duration of observation period and timing of any follow-up assessments (if appropriate). We noted any statistical measures of association between potential predictors and response status (e.g. correlations, odds ratios, etc.…), the magnitude of any associations, and whether the analyses were uni- or multi-variate, were adjusted for covariates (e.g. sex), and/or if they examined confounders, etc. If summary statistics were not reported, the researchers requested raw data from the original authors.

### Quality assurance (QA) assessment

Quality of included studies was assessed independently by three raters (JS, DGL and BE) using the 14-item Quality Assessment Tool for Observational Cohort and Cross-Sectional Studies (available at https://www.nhlbi.nih.gov/health-topics/study-quality-assessment-tools). Assessors reviewed and critically appraised each publication and differences in their independent ratings were resolved by consensus. The final ratings provided a total score (range 0–14) and a quality grading (good, fair or poor).

### Qualitative synthesis of findings

Findings were summarized from all eligible studies and we generated a list of socio-demographic and clinical variables that were reported in at least two studies undertaken in independent datasets. Each study was then assessed separately and any factor associated with a good outcome to Li was labelled as GO (Good Outcome), whilst those associated with poor outcome were labelled as PO (Poor Outcome). If the analysis of a factor in an individual study showed no statistical association with GO or PO, it was labelled as UA (uncertain association with Li response status). By the term ‘outcome’, we refer to the primary outcome that has been defined in each study (for examples: score at the Alda scale for retrospective studies if this scale was used, morbidity index before/after lithium initiation for other retrospective studies, time to relapse or presence of a relapse during follow-up for prospective studies, among different types of response assessment).

We then estimated the percentage of studies in which a factor was classified as GO, UA, or PO (i.e. number of studies in which factor was classified/total number of studies measuring that factor). The relative proportions of studies identifying a given factor respectively as GO, UA and PO were then plotted for all factors in a ‘100% stacked column’ chart. Three schematic diagrams were produced, one included all relevant studies regardless of QA assessment, the second selected studies graded as good quality only and the third selected only prospective studies. For the second and third schematic diagrams, we applied the same threshold for inclusion of a factor as used for the first schematic, namely that it was measured in at least two studies.

Lastly, we assessed concordance regarding the valence of any putative predictors by assessing the level of agreement (regarding factors associated with GO, PO or UA) according to study quality grading (categorized as Good versus Fair or Poor (combined into one category)) or study design (categorized as Retrospective versus Prospective). We considered that a factor gave concordant results when it was associated with the same type of response (GO, PO or UR) in more than 50% of independent studies. We then calculated the proportion of concordance: number of ‘concordant factors’ divided by the number of factors being studied.

## Results

### Selected articles

The initial literature search identified 3402 articles, of which 70 met criteria for full text review (see flow-chart in Fig. 1). Following review, 34 publications derived from 31 independent datasets were eligible for inclusion in the systematic review.

### Population and study design

Key characteristics of the methodology, design and samples from the included studies are presented in Additional file 2: Table S3. Location of publication varied widely, but 13 of the 34 studies were undertaken in Europe (38%). The median sample size was 112 but again varied widely (n = 21 to 4714, total sample size = 12.602), while seven studies (20%) included < 50 individuals. About 20% of studies included only individuals with a diagnosis of BD-I, whilst eleven of the studies did not specify the distribution of BD subtypes. The prevalence of females ranged from 29 to 79%, although the majority of samples included more females than males.

Twenty-five studies used a retrospective design; of these 11 studies assessed Li response using the Alda scale (Kapur et al. 2019; Saito et al. 2017; Etain et al. 2016; Sportiche et al. 2017; Scott et al. 2017; Silva et al. 2016; Rybakowski et al. 2013; Garnham et al. 2007; Grof et al. 2002; Martinsson et al. 2013; Guloksuz et al. 2012), whilst two community cohort studies used change in illness activity (e.g. readmission rates) (Kessing et al. 2011; Kessing et al. 2014). The 14 other retrospective studies employed a variety of assessments of outcome of prophylaxis ranging from number of relapses over a given time period through to change in Clinical Global Improvement (CGI) or Global Assessment Scale (GAS) scores (Shan et al. 2016; Tharoor et al. 2013; Rybakowski et al. 2009; Ozyildirim et al. 2010; Masui et al. 2008; Rybakowski et al. 2007; Washizuka et al. 2003; Tondo et al. 2001; Yazici et al. 1999; O’Connell et al. 1991; Okuma 1993). Nine studies were categorized as prospective, of which eight reported only prospective outcomes of Li treatment such as change in number of BD episodes by polarity, overall change in illness burden before/after lithium initiation for example (Post et al. 2016; Cakir et al. 2016; Kato et al. 2000; Gasperini et al. 1993; Maj et al. 1998; Degenhardt et al. 2012; Denicoff et al. 1997; Stefos et al. 1996; Kusalic et al. 1998), whilst one study reported both retrospective and prospective outcome assessments (Kulhara et al. 1999). As described in Additional file 2: Table S3, 16 studies did not report serum lithium levels while 18 studies did.

### Quality assessment of eligible studies

The median quality rating score was 7/14. However, quality gradings revealed that, whilst 11 studies (33% - n = 9.981) were graded as good or borderline good quality, 10 studies (30% - n = 2.621) were graded as poor or borderline poor (see Additional file 3: Table S1). There were recurring design and methodological weaknesses that could bias reported study findings including e.g. reliance on small and/or convenience samples, case note assessment of outcome to Li treatment by a single, unblinded investigator (without corroboration or review by another rater), failure to systematically assess putative predictors using reliable and valid tools (e.g. reliance on patient reports to assess family history of BD).

### Putative predictors of outcome to lithium treatment

Although a total of 43 potential predictors of outcome to Li were reported at least in one study, only 32 of these factors were eligible for the review (for a detailed summary of factors, see Additional file 4: Table S2). The three most frequently examined factors were: age at onset of BD (n = 22 studies), gender (n = 19) and current age/age at study inclusion (n = 17). Six other factors were examined in > 30% studies: psychotic symptoms, illness duration of BD, BD subtype, family history of BD, rapid cycling and lifetime number of mood episodes.

**Fig. 2:**
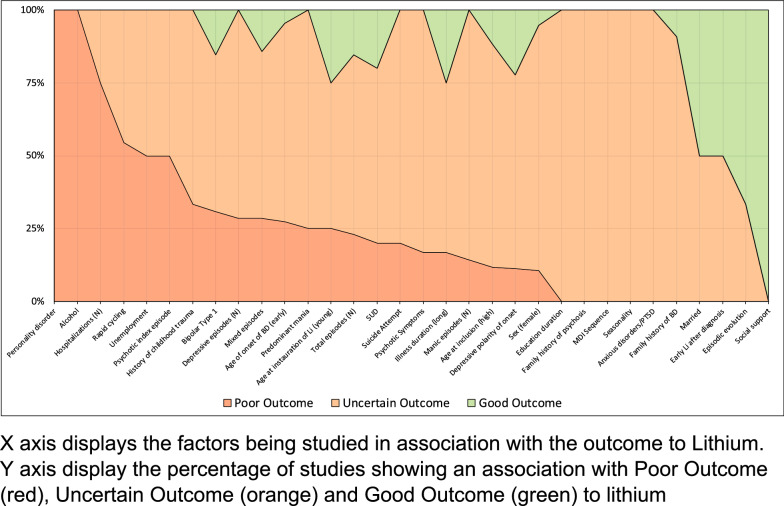
Diagram graph of all potential predictors being studied in at least 2 studies

As shown in Fig. 2, six factors were reported to be associated with either PO or GO status. Factors associated with PO were: comorbid alcohol use disorder (reported in 2 out of 2 studies); comorbid personality disorders (2/2 studies); higher lifetime number of hospitalizations (PO = 6/8; UA = 2/8 studies); and rapid cycling pattern (PO = 6/11; UA = 5/11 studies). Two factors were associated with GO: good social support (2/2 studies) and episodic (i.e. sequence of mood episodes being separated by clear periods of euthymia) evolution of illness (GO = 2/3; UA = 1/3 studies).

The other 26 factors (i.e. 81% of all factors examined) repeatedly failed to show any statistically significant positive or negative association with Li treatment (and were mainly classified as UA). Among these, five factors were classified as UA in all the eligible studies in which the factor was examined: seasonality (2/2 studies), MDI sequence (3/3 studies), time in education (5/5 studies), family history of psychosis (2/2 studies), and presence of any anxiety disorders (including PTSD) (4/4 studies).

**Fig. 3:**
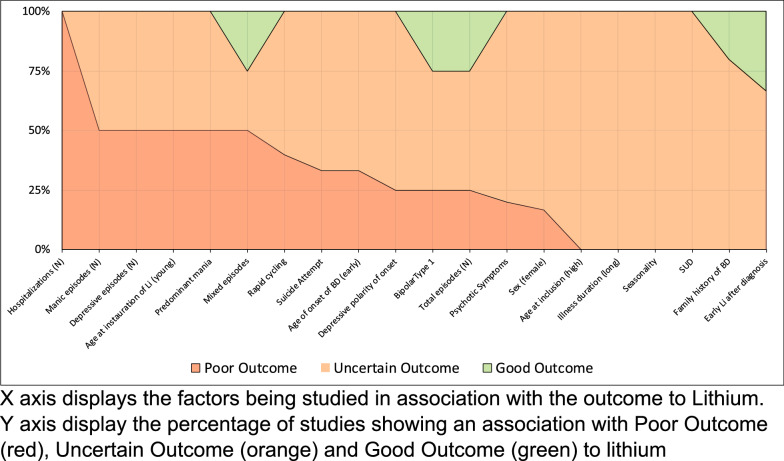
Diagram graph of all potential predictors being studied in at least 2 studies, only in good or borderline good quality studies

When the synthesis of findings was limited to the highest (good or borderline good) quality studies (11 studies; 9 independent datasets), only higher lifetime number of hospitalizations remained associated with PO and no associations were maintained for GO (see Fig. 3). When the descriptive analysis was restricted to only good quality studies, some variables being associating to PO or GO were lost (personality disorders, alcohol misuse, social support, episodic evolution and rapid cycling). This was mainly due to an insufficient number of (good quality) studies reporting these variables (personality disorders, alcohol misuse, social support, episodic evolution). For rapid cycling, it was observed that 2 of 5 good quality studies reported an association with PO, while 4 of 6 studies (fair to poor quality) reported such an association. This observation makes it possible that for rapid cycling, the quality of the studies may impact on the reports of the potential associations to PO.

**Fig. 4:**
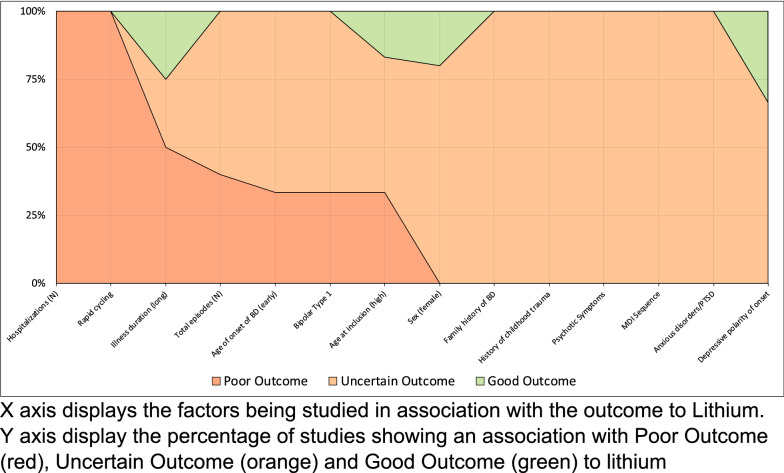
Diagram graph of all potential predictors being studied in at least 2 studies, only in prospective studies

As shown in Fig. 4, when the synthesis of findings was limited to prospective studies (n = 9), we noted that rapid cycling and number of hospitalizations were associated with PO, but no associations were maintained for GO.

Regarding concordance, we found that the level of agreement between studies regarding the predictive utility of a factor (and the valence of the association) was lower in higher quality studies (concordance rates: Higher versus Lower Quality = 65% versus 85%) and in prospective studies (concordance rates: Retrospective versus Prospective Design = 92% versus 86%).

## Discussion

After decades of research into clinical predictors of response to Li prophylaxis in BD, our disappointing findings highlight the lack of consistency across individual studies. The conclusions of this systematic review can be deduced simply by examining the dwindling number of factors associated with GO and PO status when we focus on higher quality studies or prospective designs (Figs. 1, 2 and 3). One of the most obvious conclusions is that, despite many decades of research, there remains a substantial gap in our understanding of predictors of outcome of lithium treatment indicating there is a need of high quality research on large representative samples. We do not underestimate the difficulties faced by researchers in this field and recognize the impossibility of perfect studies and the complexity of undertaking very high quality projects on this topic. However, a key message from this review is that this remains an unanswered question. The other obvious point is that there is a potential problem in undertaking further qualitative or quantitative reviews as they are all faced with the difficulties of deriving reliable and valid conclusions regarding predictors when synthesizing findings from the existing literature. This is perhaps best illustrated by the fact that higher quality or prospective studies (theoretically more reliable and/or valid) show a somewhat lower concordance (i.e. less agreement) regarding the best predictors than lower quality or retrospective studies. It is also noteworthy that meta-analyses cannot solve this conundrum, as a lack of high quality studies (compared to the number of fair/poor quality studies) is a recognized contra-indication to use that strategy or an indication to being cautious in the application of any findings in such circumstances.

Regarding specific outputs of this review, we highlight that 26 out of 32 putative predictors (selected on the basis that they were examined in at least two studies from independent datasets) failed to show any significant association with either GO or PO status. Further, the six factors identified as associated with either GO (n = 2) or PO (n = 4) did not manifest a robust association with Li response when the synthesis of data focused on studies selected for their higher quality (good or borderline fair/good) or optimal design (prospective follow-up). Interestingly, the two factors identified as being associated with GO in all the studies in which they were examined (good social support and episodic evolution of illness) were only assessed on five occassions in total and were the factors most likely to be lost from the list of predictors when studies were stratified by quality or design—partly because there were no high quality studies available assessing, for example,“good social support”. The majority of the variables associated with PO were no more robust, with the exception of higher number of prior hospitalizations and the mainly consistent evidence of poor outcome in those with rapid cycling BD in prospective studies.

Overall, the most relevant clinical implication of this review is that clinicians might have marginally more confidence in stating which factors are associated with poor outcome to Li prophylaxis, but very little certainty about the factors that are associated with good outcome. As higher lifetime number of hospitalizations consistently predicted poor outcome in the overall analyses, in the highest quality studies and in the prospective studies, another clinical implication from the review might be that lithium should be offered early in the course of bipolar disorder as also concluded in the study by Kessing et al. 2014 specifically investigating this issue. Whilst this is disappointing at one level, the clinical translation of the findings, especially if this trend continues in future studies and high quality systematic reviews, is that psychiatrists may eventually be able to avoid asking some individuals with BD to participate in a lengthy trial of Li when the clinical history reveals the presence of the factors most robustly associated with poor outcome (as they are unlikely to show any significant health gain). Unfortunately, we are still far away from being confident about this assertion, and it may well be that poor outcome associated with higher lifetime number of hospitalizations may have nothing to do with lithium in particular but may simply be a marker for poor general outcome (to psychopharmacological treatment) in patients with bipolar disorders. This is suggested by a randomized controlled study comparing lithium and lamotrigine (Licht et al. 2010). In this study the number of previous episodes (and rapid cycling), independently of treatment, was associated with poor outcome, which also has been found by others (Perlis et al. 2006).

Several factors that even meta-analytic studies have suggested may be associated with good outcome lacked a clear signal for predicting Li response in this review. Indeed, when the current review is compared with the most recent meta-analysis (Hui et al. 2019), the only findings in common are the association of poor outcome with rapid cycling BD or number of prior hospitalizations (but the strength of the reported associations was reversed between these two reviews). In part, this may be due to differences in inclusion and exclusion criteria as, for example, there was no overlap in the studies selected regarding the predictor “MDI sequence” between this review and the recent meta-analysis (Hui et al. 2019). In addition very few of the studies (e.g. Etain et al. 2017; Scott et al. 2017) selected for this review as well as the recent meta-analysis (Hui et al. 2019) studied the possibility of interdependence/interaction between predictors. Moreover, several of the included studies (n = 16) did not report lithium plasma levels in the studied samples, nor comparisons of lithium plasma level by outcome status (n = 11). Among the 7 remaining studies, the subdivision of lithium concentrations, associated with the various outcomes, was so inconsistent that this fact alone made it impossible to include lithium concentrations as a predictive factor in our systematic review. However, lithium serum levels in a range above a lower threshold around 0.45/0.60 and up to 0.80/1.00 mmol/L have been associated with superior efficacy compared to lower lithium serum levels in the maintenance treatment of bipolar disorders (Nolen et al. 2019). All of this presents a dilemma for clinicians seeking answers from the literature. Do they follow the evidence from a meta-analysis that the authors themselves report to be sub-optimal, or do they ignore even the weak signal regarding outcome prediction to lithium treatment because a rigorous review failed to find sufficient support for the factor in question? At this point of time, it seems prudent to suggest clinicians, when there are no contraindications, to offer a trial of lithium prophylaxis as the gold standard maintenance treatment in bipolar disorders to all of their patients with theses disorders, irrespective of the presence or absence of predictors potentially associated with good or poor outcome to long-term treatment with lithium.

### Perspectives and recommendations

The search for clinical predictors of response to Li remains inconclusive and unconvincing. There has been a trend for reviews and meta-analyses of existing studies, but many of these are flawed (e.g. low quality or fail to employ reliable assessments or multivariate analyses). It is perhaps more important to encourage high quality large scale studies, rather than undertaking further reviews or pooled analyses. Despite the longevity of use of Li in clinical practice, more research is required of clinically representative samples (with adequate statistical power) that employ modern analytic techniques and consider co-associations between putative predictors, including serum lithium levels, to identify individual demographic or clinical factors associated with GO or PO or specific combinations of factors or key variables underlying Li response (Passos et al. 2019; Nunes et al. 2020).

A comprehensive, reliable and valid clinical assessment needs to be incorporated into any future studies since some potential predictors of good/poor outcome to Li remain understudied (familial response to Li, alcohol use disorder, personality disorders, etc.), and/or are poorly operationalized (MDI sequence, childhood maltreatment, etc.) (Scott et al. 2018).

Although there is increasing research on potential biomarkers or biosignatures of Li response (e.g. RLiNK, https://rlink.eu.com/ in which most authors are involved, other on-going longitudinal studies or registry-based studies), they are likely to be used as well as, rather than instead of clinical factors (if any) (Hui et al. 2019; Scott et al. 2019), so an integrated science approach including rigorous clinical phenotyping remains a cornerstone of any biological or genetic research in this field (Scott et al. 2018).

## Conclusions

This systematic review of publications from the last three decades of research in the field of predictors of Li response failed to identify predictors of outcome to Li prophylaxis, except lifetime number of hospitalizations, that remained robust when the synthesis of findings was restricted to higher quality or prospective studies. Hence, it is suggested from the review that lithium should be offered early in the course of BD. It emerged that several factors are under-researched (including some potentially associated with GO such as good social support) and/or are worthy of further investigation (such as early initiation of Li and axis II disorders and alcohol use disorders). However, we conclude that the current clinical approach, i.e. a trial of Li undertaken on a case by case basis, is still required. This is because a careful examination of the design and quality of available studies identified sufficient methodological concerns to suggest that earlier reviews and meta-analyses might have significant drawbacks. Also, further systematic reviews or meta-analyses are not easy to justify until new high quality studies in large clinically representative samples are available.

## Supplementary information

**Additional file 1:** PRISMA checklist.

**Additional file 2: Table S3.** Characterization of selected studies (n=34) and the clinical predictors of outcome to long-term treatment with lithium.

**Additional file 3: Table S1.** Summary of Quality Assessment gradings for studies meeting eligibility criteria for the systematic review.

**Additional file 4: Table S2.** Number factors investigated in >= 2 from independent datasets.

## Data Availability

Not applicable; all data used in this systematic review are derived from previous publications.

## Members of the ISBD/IGSLI task force on the treatment with lithium who critically revised the article

M Bauer (University Hospital Carl Gustav Carus, TU Dresden, Dresden, Germany); M. Berk (Deakin University, Geelong, Australia); A.F. Carvalho (Centre for Addiction & Mental Health (CAMH), University of Toronto, Toronto, Canada); K.N. Fountoulakis (Aristotle University of Thessaloniki, Thessaloniki, Greece); P. Grof (Mood Disorders Center of Ottawa, University of Toronto, Canada); L.V. Kessing (Psychiatric Center Copenhagen, Copenhagen, Denmark); U. Lewitzka (Klinik und Poliklinik für Psychiatrie und Psychotherapie, Universitätsklinikum Carl Gustav Carus, Dresden, Germany); R.W. Licht (Aalborg University Hospital, Psychiatry, and Department of Clinical Medicine, Aalborg University, Aalborg, Denmark); B. Müller-Oerlinghausen (Drug Commission of the German Medical Association, Berlin, Germany); R.E. Nielsen (Aalborg University Hospital, Psychiatry, and Department of Clinical Medicine, Aalborg University, Aalborg, Denmark); W.A. Nolen (Department of Psychiatry, University Medical Center Groningen, University of Groningen, Groningen, the Netherlands); I. Pacchiarotti (Bipolar Disorder Program, Hospital Clinic, University of Barcelona, IDIBAPS, CIBERSAM, Barcelona, Spain); J.K. Rybakowski (Poznan University of Medical Sciences, Poznan, Poland); C. Simhandl (Medical Faculty, Bipolar Center, Sigmund Freud Private University, Wiener Neustadt, Austria); T. Suppes (Department of Psychiatry & Behavioral Sciences, Stanford University School of Medicine, Palo Alto, USA); E. Vieta (Bipolar Disorder Program, Hospital Clinic, University of Barcelona, IDIBAPS, CIBERSAM, Barcelona, Spain); A.H. Young (Department of Psychological Medicine, Institute of Psychiatry, Psychology and Neuroscience, King’s College London, London, UK); C. Zarate (National Institute of Mental Health (NIMH), Bethesda, Maryland, USA).

## References

[CR1] Cakir S, Tasdelen Durak R, Ozyildirim I, Ince E, Sar V (2016). Childhood trauma and treatment outcome in bipolar disorder. J Trauma Dissociation.

[CR2] Collins PY, Patel V, Joestl SS, March D, Insel TR, Daar AS (2011). Scientific Advisory Board and the Executive Committee of the Grand Challenges on Global Mental Health, et al. Grand challenges in global mental health. Nature.

[CR3] Degenhardt EK, Gatz JL, Jacob J, Tohen M (2012). Predictors of relapse or recurrence in bipolar I disorder. J Affect Disord.

[CR4] Denicoff KD, Smith-Jackson EE, Disney ER, Ali SO, Leverich GS, Post RM (1997). Comparative prophylactic efficacy of lithium, carbamazepine, and the combination in bipolar disorder. J Clin Psychiatr.

[CR5] Etain B, Lajnef M, Brichant-Petitjean C, Geoffroy PA, Henry C, Gard S, Kahn JP, Leboyer M, Young AH, Bellivier F (2017). Childhood trauma and mixed episodes are associated with poor response to lithium in bipolar disorders. Acta Psychiatr Scand.

[CR6] Garnham J, Munro A, Slaney C, Macdougall M, Passmore M, Duffy A, O’Donovan C, Teehan A, Alda M (2007). Prophylactic treatment response in bipolar disorder: results of a naturalistic observation study. J Affect Disord.

[CR7] Gasperini M, Scherillo P, Manfredonia MG, Franchini L, Smeraldi E (1993). A study of relapses in subjects with mood disorder on lithium treatment ». Eur Neuropsychopharmacol.

[CR8] Goodwin GM, Haddad PM, Ferrier IN, Aronson JK, Barnes TRH, Cipriani A, Coghill DR (2016). Evidence-based guidelines for treating bipolar disorder: revised third edition Recommendations from the British Association for Psychopharmacology. J Psychopharmacol.

[CR9] Gore FM, Bloem PJN, Patton GC, Ferguson J, Joseph V, Coffey C, Sawyer SM, Mathers CD (2011). Global burden of disease in young people aged 10-24 years: a systematic analysis. Lancet.

[CR10] Grof P, Duffy A, Cavazzoni P, Grof E, Garnham J, MacDougall M, O’Donovan C, Alda M (2002). Is response to prophylactic lithium a familial trait?. J Clin Psychiatr.

[CR11] Guloksuz S, Altinbas K, Aktas Cetin E, Kenis G, Bilgic Gazioglu S, Deniz G, Timucin Oral E, van Os J (2012). Evidence for an association between Tumor Necrosis Factor-alpha levels and lithium response. J Affect Disord.

[CR12] Hui TP, Kandola A, Shen L, Lewis G, Osborn DPJ, Geddes JR, Hayes JF (2019). A systematic review and meta-analysis of clinical predictors of lithium response in bipolar disorder. Acta Psychiatr Scand.

[CR13] Kapur V, Nadella RK, Sathur Raghuraman B, Saraf G, Mishra S, Srinivasmurthy N, Jain S, Del Zompo M, Viswanath B (2019). Clinical factors associated with lithium treatment response in bipolar disorder patients from India. Asian J Psychiatr.

[CR14] Kato T, Inubushi T, Kato N (2000). Prediction of lithium response by 31P-MRS in bipolar disorder. The International Journal of Neuropsychopharmacology..

[CR15] Kessing LV, Vradi E, Andersen PK (2016). Nationwide and population-based prescription patterns in bipolar disorder. Bipolar Disord.

[CR16] Kessing LV (2000). Lithium as the drug of choice for maintenance treatment in bipolar disorder. Acta Psychiatr Scand.

[CR17] Kessing LV, Bauer M, Nolen WA, Severus E, Goodwin GM, Geddes J (2018). Effectiveness of maintenance therapy of lithium vs other mood stabilizers in monotherapy and in combinations: a systematic review of evidence from observational studies. Bipolar Disord.

[CR18] Kessing LV, Hellmund G, Andersen PK (2011). Predictors of excellent response to lithium: results from a nationwide register-based study. Int Clin Psychopharmacol.

[CR19] Kessing LV, Vradi E, Andersen PK (2014). Starting lithium prophylaxis early v late in bipolar disorder. Br J Psychiatr.

[CR20] Kessing LV, Vradi E, Andersen PK (2015). Life expectancy in bipolar disorder. Bipolar Disord.

[CR21] Kleindienst N, Engel R, Greil W (2005). Which clinical factors predict response to prophylactic lithium? A systematic review for bipolar disorders. Bipolar Disord.

[CR22] Kulhara P, Basu D, Mattoo SK, Sharan P, Chopra R (1999). Lithium prophylaxis of recurrent bipolar affective disorder: long-term outcome and its psychosocial correlates. J Affect Disord.

[CR23] Kusalic M, Engelsmann F (1998). Predictors of lithium treatment responsiveness in bipolar patients. A two-year prospective study. Neuropsychobiology.

[CR24] Liberati A, Altman DG, Tetzlaff J, Mulrow C, Gøtzsche PC, Ioannidis JPA, Clarke M, Devereaux PJ, Kleijnen J, Moher D (2009). The PRISMA statement for reporting systematic reviews and meta-analyses of studies that evaluate healthcare interventions: explanation and elaboration. BMJ.

[CR25] Licht RW, Nielsen JN, Gram LF, Vestergaard P, Bendz H (2010). Lamotrigine versus lithium as maintenance treatment in bipolar I disorder: an open, randomized effectiveness study mimicking clinical practice The 6th trial of the Danish University Antidepressant Group (DUAG-6). Bipolar Disord.

[CR26] Maj M, Pirozzi R, Magliano L, Bartoli L (1998). Long-term outcome of lithium prophylaxis in bipolar disorder: a 5-year prospective study of 402 patients at a lithium clinic. Journal Psychiatr.

[CR27] Maj M, Pirozzi R, Starace F (1989). Previous pattern of course of the illness as a predictor of response to lithium prophylaxis in bipolar patients. J Affect Disord.

[CR28] Malhi GS, Outhred T, Morris G, Boyce PM, Bryant R, Fitzgerald PB, Hopwood MJ (2018). Royal Australian and New Zealand college of psychiatrists clinical practice guidelines for mood disorders: bipolar disorder summary. Medical J Australia.

[CR29] Martinsson L, Wei Y, Xu D, Melas PA, Mathé AA, Schalling M, Lavebratt C, Backlund L (2013). Long-term lithium treatment in bipolar disorder is associated with longer leukocyte telomeres. Translational Psychiatr.

[CR30] Masui T, Hashimoto R, Kusumi I, Suzuki K, Tanaka T, Nakagawa S, Suzuki T (2008). A possible association between missense polymorphism of the breakpoint cluster region gene and lithium prophylaxis in bipolar disorder. Prog Neuropsychopharmacol Biol Psychiatry.

[CR31] Miura T, Noma H, Furukawa TA, Mitsuyasu H, Tanaka S, Stockton S, Salanti G (2014). Comparative efficacy and tolerability of pharmacological treatments in the maintenance treatment of bipolar disorder: a systematic review and network meta-analysis. Lancet Psychiatr.

[CR32] Moher D, Shamseer L, Clarke M, Ghersi D, Liberati A, Petticrew M, Shekelle P, Stewart LA, PRISMA-P Group (2015). Preferred reporting items for systematic review and meta-analysis protocols (PRISMA-P) 2015 Statement. Syst Rev.

[CR33] Montlahuc C, Curis E, Grillault Laroche D, Bagoe G, Etain B, Bellivier F, Chevret S (2019). Response to lithium in patients with bipolar disorder: what are psychiatrists’ experiences and practices compared to literature review?. Pharmacopsychiatry.

[CR34] NIH Quality Assurance Guidelines. Study quality assessment tools, National Heart, Lung, and Blood Institute (NHLBI). 2019. https://www.nhlbi.nih.gov/health-topics/study-quality-assessment-tools.

[CR35] Nolen WA, Licht RW, Young AH, Malhi GS, Tohen M, Vieta E, Kupka RW, Zarate C, Nielsen RE, Baldessarini RJ, Severus E (2019). ISBD/IGSLI Task Force on the treatment with lithium What is the optimal serum level for lithium in the maintenance treatment of bipolar disorder? A systematic review and recommendations from the ISBD/IGSLI Task Force on treatment with lithium Version 2. Bipolar Disord.

[CR36] Nunes A, Ardau R, Berghöfer A, Bocchetta A, Chillotti C, Deiana V (2020). Prediction of lithium response using clinical data. Acta Psychiatr Scand.

[CR37] O’Connell RA, Mayo JA, Flatow L, Cuthbertson B, O’Brien BE (1991). Outcome of bipolar disorder on long-term treatment with lithium. Br J Psychiatr.

[CR38] Okuma T (1993). Effects of carbamazepine and lithium on affective disorders. Neuropsychobiology.

[CR39] Ozyildirim I, Cakir S, Yazici O (2010). Impact of psychotic features on morbidity and course of illness in patients with bipolar disorder. European Psychiatry.

[CR40] Passos IC, Ballester PL, Barros RC, Librenza-Garcia D, Mwangi B, Birmaher B (2019). Machine learning and big data analytics in bipolar disorder: a position paper from the International Society for Bipolar Disorders Big Data Task Force. Bipolar Disord.

[CR41] Perlis RH, Ostacher MJ, Patel JK, Marangell LB, Zhang H, Wisniewski SR (2006). Predictors of recurrence in bipolar disorder: primary outcomes from the Systematic Treatment Enhancement Program for Bipolar Disorder (STEP-BD). Am J Psychiatry.

[CR42] Post RM, Leverich GS, Kupka R, Keck PE, McElroy SL, Altshuler LL, Frye MA (2016). Clinical correlates of sustained response to individual drugs used in naturalistic treatment of patients with bipolar disorder. Compr Psychiatry.

[CR43] Rybakowski JK (2014). Factors Associated with Lithium Efficacy in Bipolar Disorder ». Harvard Rev Psychiatr.

[CR44] Rybakowski JK, Dembinska D, Kliwicki S, Akiskal KK, Akiskal HH (2013). TEMPS-A and long-term lithium response: positive correlation with hyperthymic temperament. J Affect Disord.

[CR45] Rybakowski JK, Permoda-Osip A, Borkowska A (2009). Response to prophylactic lithium in bipolar disorder may be associated with a preservation of executive cognitive functions. Eur Neuropsychopharmacol.

[CR46] Rybakowski JK, Suwalska A, Skibinska M, DmitrzakWeglarz M, Leszczynska-Rodziewicz A, Hauser J (2007). Response to lithium prophylaxis: interaction between serotonin transporter and BDNF Genes American Journal of Medical Genetics Part B. Neuropsychiatric Genetics.

[CR47] Saito S, Fujii K, Ozeki Y, Ohmori K, Honda G, Mori H, Kato K (2017). Cognitive function, treatment response to lithium, and social functioning in Japanese patients with bipolar disorder. Bipolar Disord.

[CR48] Schou M (1982). Trends in lithium treatment and research during the last decade. Pharmacopsychiatria.

[CR49] Scott J, Etain B, Bellivier F (2018). Can an integrated science approach to precision medicine research improve lithium treatment in bipolar disorders?. Frontiers Psychiatr.

[CR50] Scott J, Geoffroy PA, Sportiche S, Brichant-PetitJean C, Gard S, Kahn JP, Azorin JM, Henry C, Etain B, Bellivier F (2017). Cross-validation of clinical characteristics and treatment patterns associated with phenotypes for lithium response defined by the Alda scale. J Affect Disord.

[CR51] Scott J, Hidalgo-Mazzei D, Strawbridge R, Young AH, Resche-Rigon M, Etain B, Andreassen OA (2019). Prospective cohort study of early biosignatures of response to lithium in bipolar I disorders: overview of the H2020-funded R-LiNK initiative. Int J Bipolar Disord.

[CR52] Severus E, Taylor MJ, Sauer C, Pfennig A, Ritter P, Bauer M, Geddes JR (2014). Lithium for prevention of mood episodes in bipolar disorders: systematic review and meta-analysis. Int J Bipolar Disord.

[CR53] Shan GW, Makmor-Bakry M, Omar MS (2016). Long term use of lithium and factors associated with treatment response among patients with bipolar disorder. Psychiatria Danubina.

[CR54] Silva, LF, Cunha Loureiro J, Raposo Franco SC, de Lima Santos M, Secolin R, Lopes-Cendes I, de Rosalmeida Dantas C, et al. 2016. Assessing treatment response to prophylactic lithium use in patients with bipolar disorder. Jornal Brasileiro de Psiquiatria.2016;65 (1):9-16. 10.1590/0047-2085000000097.

[CR55] Sportiche S, Geoffroy PA, Brichant-Petitjean C, Gard S, Khan JP, Azorin JM, Henry C (2017). Clinical factors associated with lithium response in bipolar disorders. Australian New Zealand J Psychiatr.

[CR56] Stefos G, Bauwens F, Staner L, Pardoen D, Mendlewicz J (1996). Psychosocial predictors of major affective recurrences in bipolar disorder: a 4-year longitudinal study of patients on prophylactic treatment. Acta Psychiatr Scand.

[CR57] Tharoor H, Kotambail A, Jain S, Sharma PSVN, Satyamoorthy K (2013). Study of the association of serotonin transporter triallelic 5-HTTLPR and STin2 VNTR polymorphisms with lithium prophylaxis response in bipolar disorder. Psychiatr Genet.

[CR58] Thompson SG, Pocock SJ (1991). Can meta-analyses be trusted?. Lancet.

[CR59] Tighe SK, Mahon B, Potash JB (2011). Predictors of lithium response in bipolar disorder. Therapeutic AdvChronic Dis.

[CR60] Tondo L, Baldessarini RJ, Floris G (2001). Long-term clinical effectiveness of lithium maintenance treatment in types I and II bipolar disorders ». Br J Psychiatr.

[CR61] Tondo L, Alda M, Bauer M, Bergink V, Grof P, Hajek T, Lewitka U (2019). Clinical use of lithium salts: guide for users and prescribers. Int J Bipolar Disord.

[CR62] Washizuka S, Ikeda A, Kato N, Kato T (2003). Possible relationship between mitochondrial DNA polymorphisms and lithium response in bipolar disorder. Int J Neuropsychopharmacol.

[CR63] Yatham LN, Kennedy SH, Parikh SV, Schaffer A, Bond DJ, Frey BN, Sharma V (2018). Canadian Network for Mood and Anxiety Treatments (CANMAT) and International Society for Bipolar Disorders (ISBD) 2018 Guidelines for the Management of Patients with Bipolar Disorder. Bipolar Disord.

[CR64] Yazici O, Kora K, Uçok A, Tunali D, Turan N (1999). Predictors of lithium prophylaxis in bipolar patients. J Affect Disord.

