# Supplementary material for: Socio-demographic and clinical predictors of outcome to long-term treatment with lithium in bipolar disorders: a systematic review of the contemporary literature and recommendations from the ISBD/IGSLI Task Force on treatment with lithium
Source: Int J Bipolar Disord. 2020 Dec 16;8:40. doi: 10.1186/s40345-020-00203-3 (PMC7744282; doi:10.1186/s40345-020-00203-3)
Supplement: Supplementary file 2 — Additional file 2: Table S3. Characterization of selected studies (n=34) and the clinical predictors of outcome to long-term treatment with lithium. [file 40345_2020_203_MOESM2_ESM.docx]

Supplementary Table S3 : Characterization of selected studies (n=34) and the clinical predictors of outcome to long-term treatment with lithium.

| **Study** | **Year** | **Country** | **Place of enrolment** | **N** | **Age**  **(mean or median)** | **Sex ratio (% F)** | **BD type 1 (%)** | **AAO** | **Disease Duration** | **Criteria of outcome/**  **Design** | **Minimal, Median or Mean**  **Duration of treatment (months)** | **Excellent**  **Outcome (%)** | **Good Outcome (%)** | **Partial outcome**  **(%)** | **Poor Outcome (%)** | **Findings** |
| --- | --- | --- | --- | --- | --- | --- | --- | --- | --- | --- | --- | --- | --- | --- | --- | --- |
| Kapur (1) | 2018 | India | NimHNAS | 210 | 36,1 | 45,2 | 100 | 21,5 | 15,2 | Alda 2 groups (cut-off 7)  Retrospective | Min > 6  Median= 101 |  | 62,9 |  | 37,1 | Variables studied: illness duration (G), depressive polarity of onset (G), sex (U), family history of BD (U), age at inclusion (U), AAO (U), rapid cycling (U), mixed episodes (U), psychotic symptoms (U), SUD (U), anxious disorder (U), suicide attempt (U), number of total episodes (U), number of manic episodes (U), number of depressive episodes (U), family history of psychosis (U), number of hospitalizations (P).  Remark: in multivariate analysis, number of total episodes was associated with GO.  Lithium levels : not reported |
| Saito (2) | 2017 | Japan | University Hospitals | 96 | 53,1 | 42,7 | NA | ≈ 34 | ≈ 19 | Alda 2 groups  (cut-off 7)  Retrospective | NA |  | 27,1 |  | 72,9 | Variables studied: sex (U), family history of BD (U), AAO (U), age at inclusion (U), psychotic symptoms (U), education duration (U), number of total episodes (P).  Lithium levels : not reported |
| Etain (3) | 2017 | France | University Hospitals | 148 | 45,8 | 60,1 | 75,7 | NA | NA | Alda 3 groups  Retrospective | NA |  | 20,3 | 49,3 | 30,4 | Variables studied: social phobia (U), GAD (U), panic disorder (U), cannabis misuse (U), history of childhood trauma (P).  Remark: the study is performed on a lower sample than Sportiche et al., focuses on a new variable: childhood trauma and subdivides SUD in alcohol and cannabis use.  Lithium levels : not reported |
| Sportiche (4) | 2017 | France | University Hospitals | 300 | 45,4 | 60 | 80 | 25,8 | ≈ 19 | Alda 3 groups  Retrospective | Min > 6 |  | 17 | 52 | 31 | Variables studied : family history of BD (G), sex (U), AAO (U), age at inclusion (U), depressive polarity of onset (U), bipolarity type 1 (U), psychotic symptoms (U), rapid cycling (U), SUD (U), early lithium after diagnosis (U), anxious disorder (U), suicide attempt (U), seasonality (U), alcohol (P), mixed episodes (P).  Lithium levels : not reported |
| Scott (5) | 2017 | France | University Hospitals | 300 | ≈ 46,7 | 60 | 80 | 25,8 | 20,9 | Alda 3 groups  Retrospective | Min > 6 |  | 17 | 52 | 31 | Variables studied: AAO (U), BD type (U), manic onset (U), mixed episodes (U), anxiety disorders (U), Alcohol or Substance use disorder (P), family history of BD type I (G), duration of BD prior to Li (G), number of mood stabilizers prior Li (P)  Remark: the results are based on a multivariate adjusted analysis.  Lithium levels : not reported |
| Shan (6) | 2016 | Malaysia | Tertiary care hospital | 47 | ≈ 42 | 59,6 | 76,6 | ≈ 23 | ≈ 19 | Relapse rate, proportion of time spent ill, duration of mood episodes, hospitalization and suicidal rates  Retrospective | Min > 6 Median = 48 |  | 76,6 | 23,4 | | Variables studied: sex (U), family history of BD or other mental illnesses (U), type of bipolarity (U), psychotic features (U), rapid cycling (U), mixed episodes (U), predominant mania polarity (U), depressive polarity of onset (U), age at lithium start (U), age of onset (U), number of total episodes (U), number of manic episodes (U), number of depressive episodes (U), early lithium after diagnosis (U).  Remark: In multivariate analysis, predominant depressive polarity is associated with NR.  Lithium levels : not different between groups |
| Lima e silva (7) | 2016 | Brazil | Psychiatric clinics | 26 | 40 | 62,5 | NA | 24,2 | 12,8 | Alda continuous  Retrospective | > 6  Median = 24 |  | NA |  | NA | Variables studied: current or previous use of tobacco (G), sex (U), AAO (U), number of manic episodes (U), number of depressive episodes (U), illness duration (U), suicide attempt (U), education duration (U), family history of psychosis (U), psychotic symptoms (U).  Remark: in multivariate analysis, psychotic symptoms become associated with PO, and use of tobacco remains associated with GO.  Lithium levels : not reported |
| Post (8) | 2016 | USA /  Europe | NIMH (outpatients) | 271 | NA | NA | 80 | NA | NA | Improvement on the CGI-BP  Prospective | > 6 |  | NA |  | NA | Variables studied: socio-economic status (G), history of CT (U), psychotic symptoms (U), anxious disorder (U), family history of BD (U), AAO (U), unemployment (U), bipolarity type 1 (P), rapid cycling (P), number of total episodes (P), alcohol misuse (P).  Remark: Unemployment becomes associated with PO in multivariate analysis.  Lithium levels : not reported |
| Cakir (9) | 2015 | Turkey | University Hospitals | 92 | 40,6 | 60,7 | 100 | NA | NA | No relapse with no additional ttt  Prospective | 36 |  | 58,7 |  | 41,3 | Variables studied: history of Childhood trauma (U), anxious disorder/PTSD (U).  Lithium levels : not reported |
| Kessing (10) | 2014 | Denmark | National registers | 4714 | ≈ 49 | 57 | NA | NA | NA | No hospitalization after 5 and 10 years  Retrospective | Min > 6 |  | NA |  | NA | Variables studied: early lithium after diagnosis (G).  Remark: the association remains significant in the adjusted multivariate analysis.  Lithium levels : not reported |
| Martinsson (11) | 2013 | Sweden | Outpatient unit | 130 | 51 | ≈ 55 | ≈ 87 | NA | 29 | Alda 2 groups  Retrospective | Median = 46-52 |  | 50 |  | 50 | Variables studied: mixed episodes (G), Bipolarity type 1 (G), sex (U), age at inclusion (U), duration of the disease (U), number of depressions (P), number of (hypo)manic episodes (P), rapid cycling (P).  All participants with therapeutic serum concentration (0.5–0.9mmol/l) |
| Tharoor (12) | 2013 | India | Hospital | 122 | 41,7 | 46,7 | 100 | 26,4 | ≈ 15 | No recurrence during 2 years  Retrospective | 24 |  | 40,2 | 19,6 | 40,2 | Variables studied: Age at inclusion (U), age at onset (U), number of total episodes (U), sex (U), psychotic symptoms (U), family history of BD (U), suicide (P).  Serum lithium level above 0.6mEq/l (lower in GO) |
| Rybakowski (13) | 2013 | Poland | University Hospitals (outpatients) | 71 | 59 | 70,4 | NA | NA | NA | Alda continuous  Retrospective | 60 |  | NA |  | NA | Variables studied: hyperthymic temperament (G), depressive temperament (U), cyclothymic temperament (P), anxious temperament (P).  Serum concentration of lithium (0.5-0.8mmol/l). |
| Guloksuz (14) | 2012 | Turkey | Mood disorder center  (outpatients) | 60 | ≈ 32,2 | 58,3 | NA | ≈ 22 | ≈ 10 | Alda  3 groups  Retrospective | NA |  | 28,3 | 38,3 | 33,3 | Variables studied: sex (U), age at inclusion (U), AAO (U), duration of illness (U), density of total episodes (U), number of manic episodes (U), number of depressive episodes (U), number of mixed episodes (U), number of hospitalizations (U).  Serum lithium level above 0.6mEq/l (lower in GO) |
| Degenhardt (15) | 2012 | Interna  tional | NA | 221 | NA | NA | 100 | NA | NA | Number of mood relapses  Prospective | NA |  | NA |  | NA | Variables studied: early AAO (P), rapid cycling (P).  Lithium levels : not reported |
| Kessing (16) | 2011 | Denmark | National registers | 3762 | 49,7 | 58,4 | NA | NA | NA | No hospitalization after 5 and 10 years  Retrospective | Min > 6 | 8,9 | NA |  | NA | Variables studied: SUD (U), female gender (P), depressive polarity of onset (P), number of hospitalizations (P), retirement (P).  Lithium levels : not reported |
| Rybakowski (17) | 2010 | Poland | Outpatients lithium clinic | 60 | 52,6 | 58,3 | NA | ≈ 30 | 22,2 | No relapse during Li treatment  Retrospective | 60 | 21,7 | 78,3 | | | Variables studied: sex (U), education duration (U).  Serum concentration of lithium (0.5 -0.8 mmol/l). |
| Ozyildirim (18) | 2010 | Turkey | Mood Disorder Unit | 69 | ≈ 40 | ≈ 75 | 100 | ≈ 23 | NA | No relapse or less severe/before ttt  Retrospective | 12 |  | 69,6 |  | 30,4 | Variable studied: psychotic symptoms (P).  Lithium levels : not reported |
| Masui (19) | 2008 | Japan | University Hospital | 161 | 48,2 | 52,7 | 51,5 | ≈ 35 | ≈ 13 | No relapse  Retrospective | 12 |  | 26,7 |  | 73,3 | Variables studied: Bipolarity type 1 (G), high age at inclusion (G), sex (U), duration of illness (U), young AAO (P).  Serum concentration of lithium (0.4 - 1.2 mEq/L) |
| Rybakowski (20) | 2007 | Poland | Outpatients lithium clinic | 111 | 55,2 | 61,3 | NA | 31,2 | 8,4 | No relapse during Li treatment  Retrospective | 60 | 28 |  | 49 | 23 | Variables studied: long illness duration (G), age at inclusion (U), AAO (U), number of total episodes (U).  Lithium levels : not reported |
| Garnham (21) | 2007 | Canada | University Hospitals (outpatients) | 78 | 45,7 | 66,6 | 79,5 | 32,3 | ≈ 13 | Alda 2 groups (7)  Retrospective | Min > 6 |  | 30 | 70 | | Variables studied: AAO (G), episodic evolution (G), bipolarity type 1 (P).  Lithium levels : not reported |
| Washizuka (22) | 2003 | Japan | University  Hospital | 54 | 46,3 | 68,5 | 63 | 33,2 | ≈ 13 | No relapse  Retrospective | average ≈ 48 |  | 62,9 |  | 37,1 | Variables studied: type of bipolarity (U), age at inclusion (U), family history of BD (U), psychotic symptoms (U), rapid cycling (P), female gender (P), AAO (P)  Serum lithium concentrations (0.3–1.0 mM) |
| Grof (23) | 2002 | Canada | Psychiatric clinic | 40 | 41,3 | 72 | 60 | 25,7 | ≈ 15 | Alda 2 groups (7)  Retrospective | 12 |  | 35 |  | 65 | Variables studied: family history of good outcome to lithium (G), age at inclusion (U), AAO (U), bipolarity type 1 (U).  Lithium levels : not reported |
| Tondo (24) | 2001 | Italy | Research center (outpatients) | 360 | NA | 64,7 | 60,6 | 29,2 | NA | Relapse, time spent ill, decrease of the number of episodes  Retrospective | 12 |  | 50,3 |  | 49,7 | Variables studied: number of total episodes (G), early lithium after diagnosis (G), age at lithium start (U), education duration (U), MDI sequence (U), rapid cycling (U), psychotic symptoms (U), mixed episodes (U), depressive polarity at onset (U), family history of BD (U), married (U), sex (U), early AAO (P), bipolarity type 1 (P), unemployment (P).  Serum lithium concentration : 0,61 (+/-0,13) mmol/l |
| Kato (25) | 2000 | Japan | University Hospital | 32 | ≈ 41 | 59,4 | 81,2 | ≈ 34 | ≈ 7 | No relapse  Prospective | 12 |  | 25 |  | 75 | Variables studied: sex (U), type of bipolarity (U), polarity of onset (U), psychotic Symptoms (U), age at inclusion (U), AAO (U), duration of illness (U), MDI Sequence (U).  Serum lithium concentrations (0.3–1.0 mM) |
| Kulhara (26) | 1999 | India | Lithium clinic | 118 | 41,3 | 28,8 | 75 | ≈ 33 | 8,4 | Number of mood relapses  RetrospectivoProspective | 24 |  | 49 |  | 51 | Variables studied: social support (G), number of total episodes (U), number of manic episodes (U), number of hospitalizations (U), illness duration (U), number of depressive episodes (P).  Serum lithium concentration : 0.4 and 1.2 mEq/ l |
| Yazici (27) | 1999 | Turkey | University Hospital | 141 | ≈ 40 | 61,7 | 90,1 | ≈ 25 | ≈ 15 | Affective morbidity index(2 groups)  Retrospective | 36 |  | 60,3 |  | 39,7 | Variables studied: married (G), number of total episodes (G), depressive polarity of onset (U), family history of BD (U), rapid cycling(U), episodic evolution (U), illness Duration (U), suicide attempt (U), seasonality (U), early AAO (P), bipolarity type 1 (P), mixed episodes (P), psychotic symptoms (P), number of hospitalizations (P), personality disorder (P), young age at lithium start (P), psychotic index episode (P), ratio of mania to depression (P).  Average serum lithium concentration higher in PO. |
| Maj (28) | 1998 | Italy | University Hospital | 247 | ≈ 41 | 55,9 | 100 | NA | NA | Number of relapses, time spent in hospital  (Prospective) | 60 | 38 | | 46,6 | 15,4 | Variables studied: age at inclusion (U), sex (U), family history of BD (U), psychotic index episode (U), MDI sequence (U), number of hospitalizations (P), number of total episodes (P), rapid cycling (P).  Lithium serum concentration : range of 0.5–1.0 mmol/l, higher in PO |
| Kusalic (29) | 1998 | Canada | University Hospital | 29 | 40,9 | 79,3 | NA | NA | 11,2 | Relapse  Prospective | 24 |  | 79,3 | | 20,7 | Variables studied: female gender (G), high age at inclusion (G), SUD (G), illness duration (G), family history of BD (U), predominant mania (U).  Remark: the significant variables stay significant in the multivariate analysis.  Lithium serum concentration : 0.8–1.3 mmol/l |
| Denicoff (30) | 1997 | USA | Outpatient clinic | 42 | 41,3 | 51,9 | 63,5 | NA | NA | Severity score  Prospective | 12 |  | NA |  | NA | Variables studied: young age at lithium instauration (G), depressive polarity of onset (G), sex (U), married (U), AAO (U), bipolarity type (U), number of total (U), manic (U) and depressive episodes (U), education duration (U), illness duration (P), high age at inclusion (P), number of hospitalizations (P).  Plasma lithium concentration : 0,84 (+/-0,13) mmol/l; lower in GO |
| Stefos (31) | 1996 | Belgium | UIniversity clinic | 21 | 44,1 | 66,6 | 66,6 | 28,2 | 15,9 | Relapse  Prospective | 12 |  | 52 |  | 48 | Variables studied: social support (G), AAO (U), number of total episodes (U), age at inclusion (U).  Plasma lithium concentration 0.6 to 1.2 mmol/l |
| Gasperini (32) | 1993 | Italy | Lithium clinic (outpatients) | 113 | 45,6 | 63,7 | NA | 30,5 | 14,9 | Relapse index  Prospective | 24 |  | NA |  | NA | Variables studied: sex (U), depressive polarity of onset (U), number of total episodes (U), illness duration (P), early AAO (P), high age at inclusion (P), personality disorder (P).  Plasma lithium level : 0 5 and 0.9 mEq/l |
| Okuma (33) | 1993 | Japan | National center | 108 | 47,1 | 39,8 | 77,8 | NA | NA | Number of relapses (3 groups)  Retrospective | 24 | 19 |  | 32 | 49 | Variables studied: episodic evolution (G), AAO (U), bipolarity type 1 (U), Predominant mania (U), rapid cycling (P).  Lithium levels : not reported |
| O’Connell (34) | 1991 | USA | University  Hospital  Outpatient | 248 | 52,7 | 53 | NA | 28,8 | NA | Global Assessment Scale  Retrospective | 12 |  | 39,9 | 40,7 | 19,4 | Variables studied: married (G), age at inclusion (U), AAO (U), sex (U), number of hospitalizations (P), SUD (P).  Mean serum lithium levels for the GO  patients was 0.50, compared with 0.70 for  the PO group. |

Factors were labelled as (G), (U) or (P) when there were associated respectively with Good Outcome, Uncertain Outcome or Poor Outcome to Lithium.

AAO : Age at onset ; BD : Bipolar Disorder ; CGI-BD : Clinical Global Impression for BD ; F : females ; GAD : Generalized Anxiety Disorder ; MDI : Mania Depression Interval ; N : Number ; NA = not available ; PTSD : Post-Traumatic Stress Disorder ; SUD :Substance Use Disorder ; Ttt : treatment.
