# Supplementary material for: Socio-demographic and clinical predictors of outcome to long-term treatment with lithium in bipolar disorders: a systematic review of the contemporary literature and recommendations from the ISBD/IGSLI Task Force on treatment with lithium
Source: Int J Bipolar Disord. 2020 Dec 16;8:40. doi: 10.1186/s40345-020-00203-3 (PMC7744282; doi:10.1186/s40345-020-00203-3)
Supplement: Supplementary file 3 — Additional file 3: Table S1. Summary of Quality Assessment gradings for studies meeting eligibility criteria for the systematic review. [file 40345_2020_203_MOESM3_ESM.docx]

Supplementary Table 1: Summary of Quality Assessment gradings for studies meeting eligibility criteria for the systematic review ^a^

| **Quality Grading ^b^** | **Number**  **of studies**  (total = 34) | **Number of participants**  (total = 12,602) | **1^st^ author & publication year** |
| --- | --- | --- | --- |
| **GOOD** | 3 | 8,606 cases | Kessing et al. 2014, Martinsson et al. 2013, Kessing et al. 2011 |
| **BORDERLINE GOOD**  **(i.e. Fair/Good)** | 8 | 1,375 | Etain et al. 2017, Saito et al. 2017, Scott et al. 2017, Sportiche et al. 2017, Shan et al. 2016, Tharoor et al. 2013, Degenhardt et al. 2012, Yazici et al. 1999 |
| **FAIR** | 13 | 1,743 | Kapur et al. 2018, Post et al. 2016,  Lima e Silva et al. 2016, Cakir et al. 2015, Rybakowski et al. 2013, Guloksuz et al. 2012, Garnham et al. 2007, Washizuka et al. 2003, Tondo et al. 2001, Kulhara et al. 1999, Denicoff et al. 1997, Gasperini et al. 1993, O’Connell et al. 1991 |
| **BORDERLINE FAIR**  **(i.e. Poor/Fair)** | 3 | 356 | Ozyldirim et al. 2010, Grof et al. 2002, Maj et al. 1998 |
| **POOR** | 7 | 522 | Rybakowski et al. 2010, Masui et al. 2008, Rybakowski et al. 2007, Kato et al. 2000, Kusalic et al. 1998, Stefos et al. 1996, Okuma et al. 1993 |

^a^ Ratings are based on the judgement of yes, no or not known/not applicable in answer to questions in the National Institute of Health (NIH) quality assessment tool (https://www.nhlbi.nih.gov/health-topics/study-quality-assessment-tools).

^b^ The NIH guidance states that, in general terms, a "good" grading indicates the least risk of bias, and results are considered to be valid. A "fair" study is susceptible to some bias deemed not sufficient to invalidate its results. The fair quality category is likely to be broad, so studies with this rating will vary in their strengths and weaknesses. A "poor" rating indicates significant risk of bias and the NIH guidance suggests that studies graded as poor are excluded from the body of evidence (NIH state that the only exception allowed is if there is no other evidence available, then poor quality studies could be considered).
