# Supplementary material for: Socio-demographic and clinical predictors of outcome to long-term treatment with lithium in bipolar disorders: a systematic review of the contemporary literature and recommendations from the ISBD/IGSLI Task Force on treatment with lithium
Source: Int J Bipolar Disord. 2020 Dec 16;8:40. doi: 10.1186/s40345-020-00203-3 (PMC7744282; doi:10.1186/s40345-020-00203-3)
Supplement: Supplementary file 4 — Additional file 4: Table S2. Number factors investigated in >= 2 from independent datasets. [file 40345_2020_203_MOESM4_ESM.docx]

Supplementary Table S2: Number factors investigated in >= 2 from independent datasets.

| **Factor** | **Number of**  **independent studies** |
| --- | --- |
| 1. Age of onset of Bipolar Disorder | 22 |
| 1. Sex | 19 |
| 1. Age at inclusion | 17 |
| 1. Total number of mood episodes | 13 |
| 1. Bipolar Disorder Type 1 | 13 |
| 1. Illness duration | 12 |
| 1. Psychotic symptoms | 12 |
| 1. Family history of Bipolar Disorder | 11 |
| 1. Rapid cycling | 11 |
| 1. Depressive polarity of onset | 9 |
| 1. Number of hospitalizations | 8 |
| 1. Mixed episodes | 7 |
| 1. Number of manic episodes | 7 |
| 1. Number of depressive episodes | 7 |
| 1. Education duration | 5 |
| 1. Suicide Attempt | 5 |
| 1. Substance Use Disorders | 5 |
| 1. Ratio of mania to depression (predominant mania) | 4 |
| 1. Married | 4 |
| 1. Anxious disorders/PTSD (Post Traumatic Stress Disorder) | 4 |
| 1. Age at instauration of lithium (young) | 4 |
| 1. Early lithium after diagnosis | 4 |
| 1. History of childhood trauma | 3 |
| 1. MDI (Mania/Depression/Interval) Sequence | 3 |
| 1. Episodic evolution | 3 |
| 1. Unemployment | 2 |
| 1. Social support | 2 |
| 1. Family history of psychosis | 2 |
| 1. Psychotic index episode | 2 |
| 1. Seasonal pattern | 2 |
| 1. Alcohol use disorders | 2 |
| 1. Personality disorders | 2 |
